# Efficient high-quality and high molecular weight plant DNA extraction protocol using Percoll™

**DOI:** 10.5511/plantbiotechnology.25.0908a

**Published:** 2026-03-25

**Authors:** Kanae Nishii, Michelle L. Hart, Nathan Kelso, Sadie Barber, Michael Möller

**Affiliations:** 1 Royal Botanic Garden Edinburgh, 20A Inverleith Row, Edinburgh, EH3 5LR, Scotland, UK; 2 Kanagawa University, 3-27-1 Rokukakubashi, Kanagawa-ku, Yokohama-shi, Kanagawa 221-8686, Japan

**Keywords:** high-molecular weight DNA extraction, long-read sequencing, next generation sequencing, Percoll™, *Streptocarpus*

## Abstract

Next generation long-read sequencing is a powerful approach to generate de novo genome assemblies, however it requires high-quality and high molecular weight (HMW) DNA to reach near-chromosome level assemblies. Some plants are reported as recalcitrant to high-quality HMW DNA extraction due to high levels of secondary metabolites. *Streptocarpus schliebenii* (Gesneriaceae) is one of those highly recalcitrant plants and its DNA extraction failed repeatedly with previously published protocols. Percoll™ is a silica-based colloid coated with polyvinylpyrrolidone and utilized for various aspects in phase separation including plants’ nuclei isolation for Hi-C library and DNA extraction to generate bacterial artificial chromosome clones. In this study, we developed a HMW DNA extraction protocol for long-read sequencing that included a Percoll™ gradient step. To establish a stable protocol, we examined and modified buffers and steps of several previous Percoll™ gradient protocols. Instead of the previously used agarose plug method for Percoll™ DNA extraction, CTAB lysis followed by Qiagen Genomic-Tips was employed. Three *Streptocarpus* species generated optimal quality and HMW DNA. The method was further tested in 12 species across a wide range of plant lineages. The results were species specific. While HMW DNA was obtained from seven species, HMW and high-quality DNA were obtained from four species, i.e. *Iris pseudacorus*, *Pulmonaria affinis*, *Corytoplectus speciosus*, and *Ilex aquifolium*. This indicates the wide applicability of this protocol for plants. This protocol provides a useful resource for those who are working on de novo plant genome projects of recalcitrant material to obtain optimal DNA for long-read sequencing.

Long-read next generation sequencing, so-called third generation sequencing, can produce vast amounts of long sequencing reads. The method has substantially decreased the time and cost to obtain de novo near-chromosome level genome assemblies from non-model organisms including plants and is now widely used for small and large-scale projects (e.g., Nishii et al. 2022; The Darwin Tree of Life Project Consortium 2022). Oxford Nanopore Technologies plc. (ONT, Oxford, UK) and Pacific Bioscience of California, Inc (PacBio, Menlo Park, CA, USA) are leading long-lead sequencing providers: the former can produce long reads as long as the input DNA molecule (e.g., Jain et al. 2016), the latter developed high fidelity HiFi sequencing with circularized libraries and produces more accurate long reads.

In a previous study, we reported the genome assembly of *Streptocarpus rexii* (Gesneriaceae) using the PromethION platform of ONT. For this, it was necessary to develop a high quality and high molecular weight (HMW) DNA extraction method specific for the recalcitrant *Streptocarpus* (Nishii et al. 2022). Further, we reported an updated DNA extraction method for PacBio HiFi sequencing (Nishii et al. 2023), and the resulting genome assembly was highly contiguous with near-chromosome length contigs (Nishii et al. 2023). In general, PacBio HiFi requires more higher quality and HMW DNA than ONT, with a length of “gDNA > 40 kb in size, on average” (PacBio 2022) when analyzed using pulse field gel electrophoresis using the Femto Pulse system (Agilent Technologies, Inc., Santa Clara, CA, US). The Femto Pulse associated genome quality number (GQN) indicates the quality and integrity of DNA and gives the percentage of DNA fragments above a set size threshold (e.g., a GQN_10 kb_ of 7.0 indicates that 70% of fragments are above 10 kb). The suggested optimal fragment length for PacBio HiFi input DNA are GQN_10 kb_≥7.0 and GQN_30 kb_≥5.0 (PacBio 2024a). It is also recommended to avoid guanidium thiocyanate, phenol and chloroform during DNA extraction (PacBio 2022). Further, the DNA quality should also be assessed by spectrophotometer, such as NanoDrop (Thermo Fisher Scientific, Waltham, MA, USA), with absorbance maxima at 230 nm (A230), 260 nm (A260), and 280 nm (A280). The ratios of these suggest pure high-quality DNA at A260/A280 ∼1.8, and A260/A230 ≥2.0 (Matlock 2015; PacBio 2022; Thermo Scientific 2024). The fluorometer-based DNA quantification using Qubit (Thermo Fisher Scientific) is recommended, and these concentration values should ideally be similar to those measured using a spectrophotometer, then indicating high-purity DNA. Guidelines suggest that the values should be less than 50% different between spectrophotometer and fluorometer measurements (PacBio 2022).

While DNA of *Streptocarpus* species had been successfully extracted for PacBio sequencing using a previous protocol (Nishii et al. 2023), we encountered repeated failures when working with the highly recalcitrant *Streptocarpus schliebenii* originally collected in Tanzania by SB (Supplementary Table S1), using previously reported protocol (Supplementary Table S2, KN467, KN468). Although the molecular weight was high (GQN_10 kb_ 9.5 and 9.6, and GQN_30 kb_ 7.8 and 8.0 respectively), the low A260/A230 ratios (1.27 and 1.46) suggested that the DNA extraction contained contaminants of secondary metabolites that potentially affect downstream applications. Thus, here we developed a more efficient protocol to remove the DNA contaminants to obtain high-quality and HMW DNA from the recalcitrant *S. schliebenii*. To this end, we added a Percoll™ gradient phase separation step to remove DNA contaminants such as secondary metabolites from cells and nuclei (Cytiva, Marlborough, MA, USA).

Percoll™ is a silica colloid coated with polyvinylpyrrolidone (PVP) and is used for various applications of phase separation by density centrifugation. It can separate different cell lines, different organelles, and viruses (Pertoft 2000; Pertoft et al. 1978; Wolff 1975). Percoll™ has been used to isolate plant organelles such as chloroplast (Bhattacharya et al. 2020), and nuclei (Folta and Kaufman 2006; McKeown et al. 2008; Sikorskaite et al. 2013). It was also used for DNA extraction for BAC clones (Hein et al. 2005; Peterson et al. 2000), and nuclei preparation for Hi-C chromatin conformation capture sequencing (Hilario 2019).

When developing the new protocol for *S. schliebenii*, we compared previous Percoll™ methods reported by Peterson et al. (2000), Folta and Kaufman (2006), and Sikorskaite et al. (2013), summarized in Supplementaty Table S3. Main differences between the protocols were Percoll™ concentration, buffer composition, and centrifugation force and duration (Folta and Kaufman 2006). For the later stages of DNA extraction, instead of the manual agarose plug method used in Peterson et al. (2000), CTAB lysis following a Qiagen Genomic-Tips kit (Qiagen, Hilden, Germany) was used, since it worked well in *Streptocarpus* (Nishii et al. 2023). Results of Percoll™ gradient tests indicated that using intermediate layers, such as with 60% Percoll™ layered on 2.5 M sucrose gradient, or 30% Percoll™ layered on 80% Percoll™, generated HMW and high-quality DNA but the amount obtained per sample was small, less than 50 ng DNA per gram fresh leaf sample (Supplementaty Table S4). The method from Peterson et al. (2000) using pellets in 37.5% Percoll™, resulted in higher amounts of DNA (613 ng DNA from gram fresh leaf sample), but the DNA was fragmented (GQN_10 kb_=5.8, GQN_30 kb_=1.0, Supplementaty Table S4), and thus we here present a further modified protocol.

In this protocol, we selected a sucrose-based extraction buffer to isolate less vacuolated small cells and nuclei as in Nishii et al. (2023). A hexylene glycol buffer was chosen for the Percoll™, as this combination accelerated the speed of sample throughput at the Genomic-Tips step (KN personal observations). For the Percoll™ gradient step, we employed a single layer of 37.5% Percoll™ in a hexylene glycol-based gradient buffer similar to Peterson et al. (2000) and Folta and Kaufman (2006) but with modifications: 1) MgCl_2_ was omitted and EDTA added for preventing potential DNase activities in the cell extract and downstream steps, 2) Triton X-100, which lyses cell membranes and/or increases their permeability (Cornett and Shockman 1978; Koley and Bard 2010), was added to the nuclei isolation buffer but not to the gradient buffer to prevent excess cell lysis in this step, 3) the duration of centrifugation was reduced to 10 min for each step, and 4) instead of the agarose plug DNA extraction method used in Hein et al. (2005) and Peterson et al. (2000) (detailed protocol described in Jones and Chi 1997), we used CTAB lysis and the Genomic-Tips kit (Supplementaty Table S3).

Whereas we used lysis at 58°C for 4 h previously (Nishii et al. 2023), here the lysis condition was 55°C for 30 min to produce HMW DNA. We observed that lysis at a higher temperature of 58°C for 30 min showed lower GQN values (DNA-ID: KN499, GQN_10 kb_=7.5, GQN_30 kb_=3.7, Supplementaty Table S2, Supplementary Figure S1). We further confirmed for *Streptocarpus grandis*, a previously tested species in Nishii et al. (2023), that the lysis condition at 55°C and 30 min worked well with or without Percoll™, and obtained similar amounts of DNA as previously reported (Nishii et al. 2023: 100–250 ng DNA per gram fresh leaf sample; Supplementaty Table S5: 126–328 ng DNA per gram fresh leaf sample).

To demonstrate the reproducibility of the protocol, three *Streptocarpus* species were tested in triplicates in small scale extractions (approximately 2 g fresh leaf starting material; Figure 1). The method is described here briefly, with a detailed protocol provided in Supplementary Document 1 and key steps are illustrated in Supplementary Figure S2. Approximately two grams of fresh leaf tissues were ground in liquid nitrogen with pestle and mortar. The ground sample was suspended in 50 ml of ice-cold nuclei isolation buffer (10 mM Tris-HCl pH 8.0, 10 mM EDTA pH 8.0, 500 mM sucrose, 100 mM KCl, 4 mM spermidine, 1 mM spermine, 0.1% β-mercaptoethanol) and filtered through nylon mesh of pore size 100 µm (e.g., Corning cell strainer, Corning, NY, US) into 50 ml Falcon tubes. A 5% volume of 10% Triton X-100 in nuclei isolation base buffer (10% Triton X-100, 10 mM Tris-HCl pH 8.0, 10 mM EDTA pH 8.0, 500 mM sucrose, 100 mM KCl) was added to the filtrate, gently mixed and centrifuged at 2,000×g for 10 min at 4°C. In this step, cells smaller than <100 µm in diameter and nuclei are pelleted, but cell debris and organelles left in the supernatant. The obtained nuclei & cell pellet was then suspended in 4 ml hexylene glycol gradient buffer (500 mM hexylene glycol, 10 mM PIPES-KOH pH 7.0, 10 mM EDTA, 5 mM β-mercaptoethanol). The suspension was layered on top of a layer of 5 ml 37.5% Percoll™ in hexylene glycol gradient buffer in a new Falcon tube and centrifuged at 1,200×g for 10 min at 4°C. For *Streptocarpus*, the suspension separated into a top green liquid layer, a middle whiteish liquid layer, and a bottom layered nuclei & cell pellet (Supplementary Figure S2E). Both green and white liquid layers were discarded, and the pellets suspended in 4 ml hexylene glycol gradient buffer and centrifugated at 2,000×g for 10 min at 4°C. The supernatant was decanted and discarded and the pellet then suspended in 2 ml CTAB lysis buffer (100 mM Tris-HCl pH 8.0, 20 mM EDTA pH 8.0, 1.4 M NaCl, 2% CTAB), supplemented with 1% PVPP and 8 µl RNase A (100 mg ml^−1^) and transferred to a new 2 ml Eppendorf tube. The samples were incubated in a heated block at 55°C for 5 min, before 40 µl proteinase K (Qiagen, catalog no. RP107B) was added and the samples gently mixed. They were then further incubated at 55°C but up to a potential maximum time of 30 min including the time before proteinase K addition. The after-lysis steps were carried out at room temperature unless mentioned otherwise. Lysates were centrifuged at 11,000 rpm for 10 min, the supernatant collected into a Falcon tube, the pH adjusted by adding an appropriate amount of 0.25 N HCl to reach a pH of 7.0–7.5. An equal volume of dH_2_O was added to the pH adjusted lysate (a mixture of lysate and 0.25 N HCl) and gently mixed to reduce the salt concentration. Genomic-Tips 20/G (Qiagen) DNA extraction and purification was performed following the manufacturer’s protocol. The obtained 2 ml DNA solution was mixed with 0.7 × volume isopropanol in two 2 ml tubes. After DNA precipitation at −20°C overnight, the tubes were centrifuged at 11,000 rpm for 10 min. Following a 70% ethanol wash and air drying, the DNA was eluted in 0.1 × TE buffer (1 mM Tris-HCl pH 8.0, 0.1 mM EDTA) at 50°C 300 rpm for one hour. Quality control of the DNA was performed using a Nanodrop spectrophotometer and a Qubit fluorometer functions in a DeNovix DS-11 machine (DeNovix Inc. Wilmington, DE, USA), and fragment size evaluation was performed with Femto Pulse.

**Figure 1:**
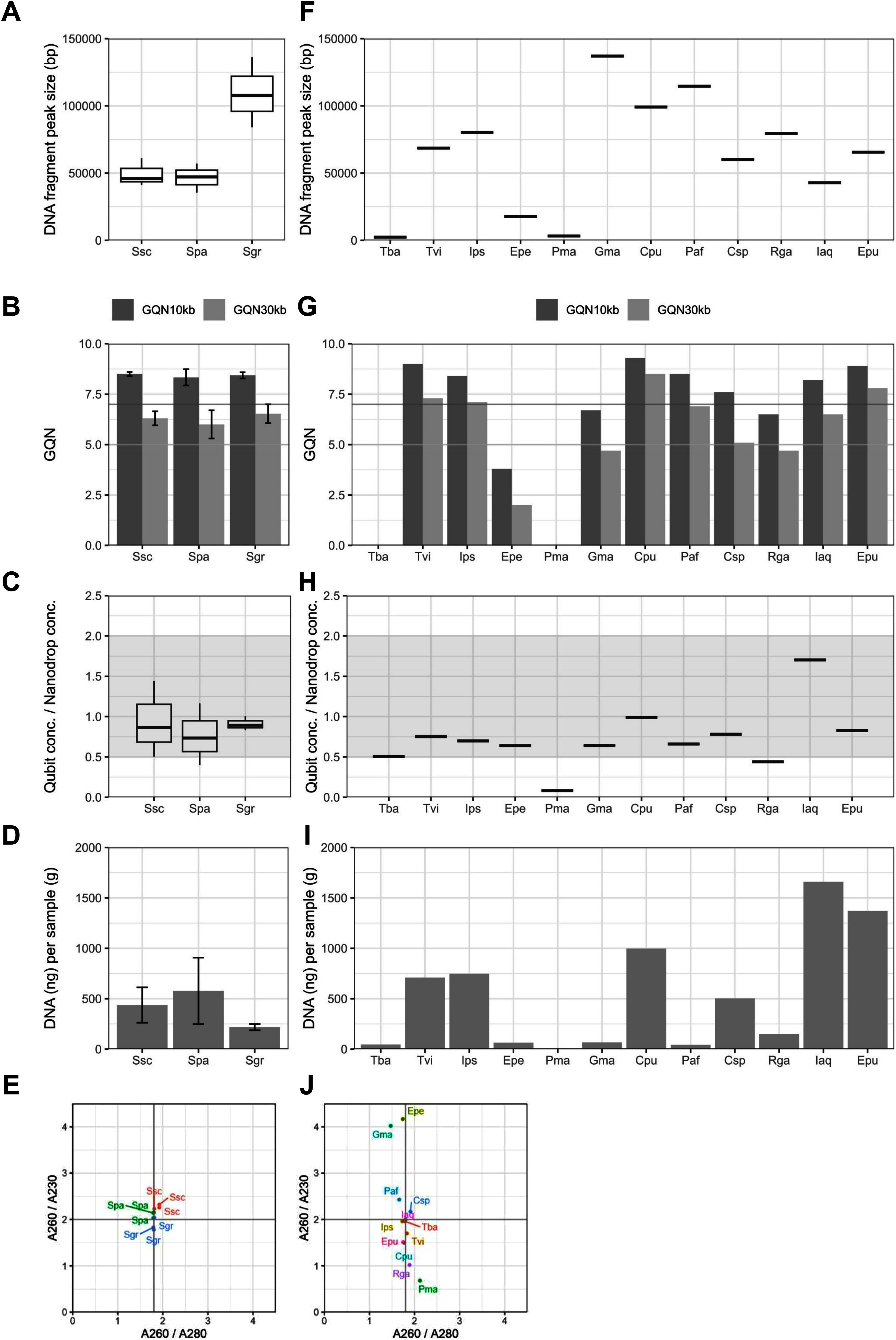
Figure 1. DNA quality and quantity obtained using the protocol developed in this study. A–E. Three *Streptocarpus species*. Error bars indicate standard deviation, *n*=3. F–J. Twelve samples across seed plants. A, F. DNA fragment peak size. B, G. Genome quality number (GQN). Gray line indicates minimum value for optimal GQN_10 kb_ (7.0) and light gray line indicates that of GQN_30 kb_ (5.0). C, H. Ratio between DNA concentrations measured by Qubit over Nanodrop. Shading indicates optimal range (0.5–2.0, i.e. <50% difference). D, I. Obtained DNA in ng per gram fresh leaf sample. E, J. Dot plot of A260/A280 ratios (x-axis) and A260/A230 ratios (y-axis). Data also listed in Supplementary Table S6. Ssc: *Streptocarpus schliebenii*, Spa: *Streptocarpus papangae*, Sgr: *Streptocarpus grandis*, Tba: *Taxus baccata* “*Fastigiata*”, Tvi: *Tulbaghia violacea*, Ips: *Iris pseudacorus*, Epe: *Epimedium perralderianum*, Pma: *Prunus maackii*, Gma: *Geranium macrorrhizum*, Cpu: *Cyclamen purpurascens*, Paf: *Pulmonaria affinis*, Csp: *Corytoplectus speciosus*, Rga: *Rhynchoglossum gardneri*, Iaq: *Ilex aquifolium*, Epu: *Erigeron pulchellus*. Graphs were generated using ggplot2 (Wickham 2016) in R v.4.4.1 (R Core Team 2020) and RStudio (Posit team 2025).

For large-scale extractions (Supplementary Table S2, KN507), we experimented with 18.2 g fresh leaf samples ground gram by gram in liquid nitrogen, suspended in 500 ml nuclei isolation buffer, filtered and the filtrate aliquoted into 50 ml Falcon tubes. The following steps were performed following the CTAB lysis step. All lysate was collected in one Falcon tube and DNA extraction and purification was performed using two Genomic-Tips 100/G columns.

The protocol presented here resulted reproducibly in DNA of high quality and high molecular weight (Figure 1, Supplementary Figure S3, Supplementary Tables S2, S5), suitable as input DNA for PacBio HiFi sequencing. The absorbance maxima ratio values for pure DNA of A260/A280 ∼1.8 and A260/A230 ≥2.0 (Matlock 2015; PacBio 2022; Thermo Scientific 2024) were satisfied by the obtained DNA (Figure 1): the peak fragment size was around 50 kbp or higher, the GQN values were all well above PacBio’s guidelines of GQN_10 kb_≥7 and GQN_30 kb_≥5 (Figure 1, Supplementary Figure S3). The concentration obtained by Qubit fluorometer and Nanodrop spectrophotometer functions of DeNovix was similar. The amount of ng DNA obtained per gram fresh leaf sample was on average 437, 578 ng and 218 ng for *S. schliebenii*, *S. papangae*, and *S. grandis* respectively. The greater variation at individual extraction level of 249.5 to 885.0 ng might be explained by the sample quality (e.g., status of individual leaves, older or younger) and perhaps sample handling during DNA extraction (e.g., part loss of pellet when removing supernatant). However, the quality and quantity values of the scaled-up DNA extraction with ∼18 g fresh leaf tissue were well in line with the smaller extractions and demonstrate the scalability of the protocol (Supplementary Table S2, KN507). A standard PacBio HiFi library preparation requires 3 µg input DNA per gigabytes of genome (PacBio 2020) or for the Revio system 2 µg per cell (PacBio 2024b). In the case of *Streptocarpus*, with an approximately 1 Gb average diploid genome size (Möller 2018), 4–12 g of leaf sample might be sufficient to obtain enough DNA for library preparation, though often sequencing facilities require more for quality control steps.

The protocol was further tested in 12 species across a wide range of seed plants (Figure 1, Supplementary Tables S1, S6). Here, seven samples (*Tulbaghia violacea, Iris pseudacorus, Cyclamen purpurascens, Pulmonaria affinis, Corytoplectus speciosus, Ilex aquifolium, Erigeron pulchellus*) generated HMW DNA that showed a high DNA fragment size peak (Figure 1F) and complied with GQN criteria (Figure 1G), three species generated slightly fragmented DNA (*Epimedium perralderianum, Geranium macrorrhizum, Rhynchoglossum gardneri*), and two (*Taxus baccata, Prinus maackii*) yielded very small amounts of highly fragmented DNA. In most species, the ratio between DNA concentration measured by Qubit and Nanodrop was not exceeding 50% difference (between 0.5 and 2.0 of “Qubit conc./Nanodrop conc.” in Figure 1H) indicating suitability for proceeding PacBio library preparation (PacBio 2022), except for *R. gardneri* with slightly low (0.43) and *P. maackii* with low (0.08) values. For six species (*T. violacea, I. pseudacorus, C. purpurascens, C. speciosus, I. aquifolium, E. pulchellus*), the amount of DNA was more than 500–1500 ng DNA per gram fresh leaf sample (Figure 1I). Of those seven species with HMW DNA, four showed an optimal DNA quality indicated by their A260/A280 and A260/A230 values, while the other three samples had low A260/A230 values, indicating that contaminants were present in the DNAs. To summarize, four in 12 tested species generated HMW and high-quality DNA.

Overall, our results indicated a high species specificity, and for example in Gesneriaceae, the protocol was successful for three *Streptocarpus* species and *C. speciosus* but not for the other Gesneriaceae *R. gardneri*. On the other hand, for four out of 12 seed plant species, including monocots, eudicots, core eudicots, the extracted DNA reached the high demands for LRS, indicating the wider applicability of this protocol. *Cyclamen purpurascens*, reported as recalcitrant (Kasajima et al. 2013) generated HMW here and most of quality scores were optimal but with a slightly low A260/A230 value. Some simple modifications, such as increased amount of nuclei extraction buffer, or repeated wash steps after the Percoll™ gradient step before cell lysis, might improve the DNA quality of the samples that failed to reach optimal quality values.

In conclusion, the Percoll™ protocol presented here successfully removed DNA contaminants from three *Streptocarpus* species including the highly recalcitrant *S. schliebenii*, as well as of a range of seed plants. The variable results across all samples points to the need of species-specific approaches, where protocols require optimization for some species. The method presented here is a useful resource for the plant science community particularly those intending to perform de novo genome sequencing from plants recalcitrant for high-quality and HMW DNA extraction.

## Abbreviations

BAC clone: bacterial artificial chromosome
CTAB: cetyltrimethylammonium bromide
EDTA: ethylenediaminetetraacetic acid
GQN: genome quality number
HMW: high molecular weight
KCl: potassium chloride
MgCl_2_: magnesium chloride
ONT: Oxford Nanopore Technologies plc.
PacBio: Pacific Bioscience of California, Inc
PVPP: polyvinylpolypyrrolidone
RBGE: Royal Botanic Garden Edinburgh

## Description of supplementary files

Supplementary Document 1. Detailed protocol developed in this study.

Supplementary Figure S1. Femto Pulse result for DNA obtained with lysis condition of 58°C for 30 min.

Supplementary Figure S2. Key steps for nuclei & cell isolation and Percoll™ gradient.

Supplementary Figure S3. Representative Femto Pulse result for *Streptocarpus schliebenii* DNA obtained using protocol established in this study.

Supplementary Table S1. Plant materials tested in this study.

Supplementary Table S2. Quality control values of *Streptocarpus* DNA extracted using different methods and conditions.

Supplementary Table S3. Comparison between protocols for nuclei isolation and/or DNA extraction using Percoll™ gradients.

Supplementary Table S4. Examples of DNA quality control results obtained during protocol development for *Streptocarpus schliebenii* using different Percoll™ gradient conditions.

Supplementary Table S5. Quality control values of DNA obtained from *Streptocarpus grandis* with or without Percoll™ and 30 min lysis time.

Supplementary Table S6. Quality control values of DNA obtained from a wide range of plants.
