# Supplementary material for: Efficient high-quality and high molecular weight plant DNA extraction protocol using Percoll™
Source: Plant Biotechnol (Tokyo). 2026 Mar 25;43(1):139–44. doi: 10.5511/plantbiotechnology.25.0908a (PMC13170785; doi:10.5511/plantbiotechnology.25.0908a)
Supplement: Supplementary Data [file plantbiotechnology-43-1-25.0908a-s001.pdf]

Supplementary Document 1

Detailed protocol developed in this study

The Percoll™ HMW DNA extraction method

Materials

Chemicals & Reagents

<Stock solutions>

- Nuclei isolation base buffer

  *Autoclave, and store in fridge

Chemical (final concentration)		Stock	In 1 L
Tris-HCl pH 8.0	(10 mM)	1 M	10 ml
EDTA pH 8.0	(10 mM)	500 mM	20 ml
Sucrose	(500 mM)	Powder	171.2 g
KCl	(100 mM)	Powder	7.5 g
dH_2O			Add to make up 1 L

- 4 M Spermidine (store in freezer)

  *Dissolve spermidine in nuclease free water. Divide small aliquots into 2 ml tubes and store at -20 °C. Avoid repeated thawing and freezing.

- 1 M Spermine (store in freezer)

  *Dissolve spermidine in nuclease free water. Divide small aliquots into 2 ml tubes and store at -20 °C. Avoid repeated thawing and freezing.

- 1 M Tris-HCl pH 8.0
- 0.5 M EDTA pH 8.0
- 0.1 × TE buffer (1 mM Tris-HCl pH8.0, 0.1 mM EDTA)
- 0.25 N HCl
- CTAB lysis buffer

  *Autoclave; can be stored at room temperature

Chemical (final concentration)		Stock	In 1 L
Tris-HCl pH 8.0	(100 mM)	1 M	100 ml
EDTA pH 8.0	(20 mM)	500 mM	40 ml
NaCl	(1.4 M)	Powder	81.8 g
CTAB	(2%, v/v)	Powder	20.0 g
dH_2O			Add to make up 1 L

- Triton X-100 working solution (10% Triton X-100 in nuclei isolation base buffer, keep in fridge)
- Buffer QBT (Qiagen #19054)
- Buffer QC (Qiagen #19055)
- Buffer QF (Qiagen #19056)

<Chemicals>

- β-mercaptoethanol
- Percoll™
- Triton X-100
- Hexylene glycol (2-methyl-2,4-pentanediol
- PIPES-KOH pH 7.0
- PVPP
- RNaseA (100 mg ml^{-1})
- Proteinase K (Qiagen, #RP107B)
- Isopropanol
- Nuclease free water
- Liquid nitrogen

<Working solutions>

*Prepare working solutions on the day of DNA extraction

- Nuclei isolation buffer

*Prepare 50 ml per 2 g fresh leaf sample

*To prepare ~100 ml buffer, add 100 µl 4 M spermidine, 100 µl 1M spermine, 100 µl β-mercaptoethanol to 100 ml nuclei isolation base buffer

- 2 × hexylene glycol gradient buffer

*Prepare 7 ml per 2 g fresh leaf sample

**Chemical (final concentration)**		**Stock**	**In 10 ml**
Hexylene glycol	(1.0 M)	100%	1.18 g
PIPES-KOH	(20 mM)	500 mM	0.4 ml
EDTA	(20 mM)	500 mM	0.4 ml
β-mercaptoethanol	(10 mM)	100%	7.8 µl
dH_2O			Add to make up 10 ml

- 1 × hexylene glycol gradient buffer

*Prepare 8 ml per 2 g fresh leaf sample

*Mix equal volumes of dH_2O and 2 × hexylene glycol gradient buffer to make up 1 × buffer

- Percoll™ gradient solution (37.5% Percoll™ in hexylene glycol gradient buffer)

*Prepare 5 ml per 2 g fresh leaf sample

*To prepare 10 ml, mix 5 ml 2 × hexylene glycol gradient buffer, 3.75 ml Percoll™, and 1.25 ml dH_2O

Consumables

- Genomic-Tips (Qiagen)
- 50 ml and 15 ml Falcon tubes
- 2 ml Eppendorf tubes
- 1.5 ml Eppendorf LoBind tubes
- Invitrogen™ Qubit™ Assay Tubes (Invitrogen™ #Q32856)
- Pipette tips for Rainin or Gilson pipettes (2 µl, 20 µl, 200 µl, 1000 µl)
- Wide orifice pipet tips (200 µl, 1000 µl)
- 3 ml disposable plastic pipettes

- pH indicator strips (e.g. Fisherbrand™ pH indicator paper sticks, Fisher Scientific UK, #10333501)
- Genomic DNA 165 kb kit (Agilent Technologies, #FP-1002-0275) for Femto Pulse analyses
- Quant-iT™ Qubit™ dsDNA HS assay kit (Thermo Fisherr Scientific, #33120) for fluorometer analyses

Equipment

- Centrifuge for 2 ml Eppendorf tubes
- Centrifuge for 50 ml Falcon tubes
- Downflow bench or fume hood
- Gilson or Rainin pipettes (2 µl, 20 µl, 200 µl, 1000 µl)
- Ceramic mortar and pestle and Styrofoam mortar holder
- Heated block
- Water bath
- Freezer and fridge
- Femto Pulse
- Nanodrop or Denovix
- Qubit or Denovix

Before starting

*All following steps where β-mercaptoethanol is involved should be performed on a downflow bench or in a fume hood

1. Prepare nuclei isolation buffer in Falcon tube or glass beaker, leave on ice
2. Prepare 2 × hexylene glycol buffer, leave on ice
3. Prepare 1 × hexylene glycol buffer, leave on ice
4. Prepare Percoll™ gradient solution, leave on ice

Procedure

*For small scale (2 g fresh leaf sample)

1. Prepare 50 ml nuclei isolation buffer in glass beaker or Falcon tube and leave on ice
2. Measure 1 g fresh leaf tissue and place in mortar, prepare a second to obtain 2 g fresh leaf tissue
3. Add liquid nitrogen to sample in mortar and grind for 6-10 seconds with pestle after liquid nitrogen had evaporated while sample is frozen. Quickly add liquid nitrogen to ground sample a second time and repeat grinding step. Repeat liquid nitrogen addition and grinding a third time until leaf tissue becomes fine powder. Never allow sample to thaw. Move powder immediately to beaker containing nuclei isolation buffer. Repeat grinding with 2nd 1 g fresh leaf sample and move ground tissue to same beaker to obtain 2 g ground leaf tissue
4. Add 5% volume of Triton X-100 working solution to ground sample suspension and gently mix by inversion
5. Centrifuge at 2,000 ×*g* for 10 minutes at 4 ^{o}C
6. Remove and discard supernatant
7. Add 4 ml 1 × hexylene glycol gradient buffer and mix with paint brush or pipet tip
8. To new Falcon tube, add 5 ml Percoll™ gradient solution. Using a 3 ml disposable plastic pipette or 1 ml wide orifice pipet tip, gently layer the sample suspension on top of Percoll™ gradient solution. When layering slowly add sample suspension drop by drop on the internal wall close to the surface of the Percoll™ gradient solution layer
9. Centrifuge at 1,200 ×g for 10 minutes at 4 ^{o}C
10. Using a 3 ml disposable plastic pipette or 1 ml wide orifice pipet tip, remove and discard the supernatant. (In the case of *Streptocarpus*, two liquid layers result and both are removed and discarded but not the pellet.)
11. Add 4 ml 1 × hexylene glycol gradient buffer and disperse pellet with fine paint brush or pipet tip
12. Centrifuge at 2,000 ×*g* for 10 minutes at 4 ^{o}C
13. Using a 3 ml disposable plastic pipette or 1 ml wide orifice pipet tip, remove and discard supernatant
14. Add 2 ml CTAB lysis buffer supplemented with 1% PVPP and 8 µl RNase A and mix with paint brush or pipet tip
15. Move sample to 2 ml Eppendorf tube using 1 ml wide orifice pipette tip

16. In heated block, incubate tube at 55°C for 5 minutes
17. Add 40 µl Proteinase K. Gently mix by inverting tube
18. Continue incubating at 55°C for ~25 minutes but not exceeding in total 30 minutes
19. Centrifuge at 11,000 rpm for 10 minutes at room temperature
20. Move supernatant to new Falcon tube (Falcon tubes are easier to handle at this stage)
21. Check pH using pH indicator strips, and adjust sample to pH 7.0 – 7.5 by adding small amounts (e.g., 50 µl each time) 0.25 N HCl each time
22. To lower the salt concentration, the volume of lysate (a mixture of lysate and 0.25N HCl) is doubled by adding dH_2O, and the solution gently mixed
23. Set up Genomic-Tips 20/G. Prepare six 15 ml Falcon tubes, and label 1) QBT, 2) sample, 3) QC1, 4) QC2, 5) QC3, and 6) QF
24. Set water bath to 50°C and prewarm buffer QF
25. Place Genomic-Tips 20/G column on 1) QBT tube. Apply 1 ml buffer QBT and allow buffer to flow through column by gravity
26. Move Genomic-Tips to 2) sample tube. Apply all sample and allow to flow through column by gravity
27. Move Genomic-Tips to 3) QC1 tube. Apply 1 ml buffer QC and allow to flow through column by gravity. Repeat for tube 4) QC2 and tube 5) QC3
28. Move Genomic-Tips to 6) QF tube. Apply 2 ml 50°C prewarmed QF buffer and allow buffer to flow through column by gravity
29. Aliquot 1 ml eluted DNA in QF buffer each to two 2 ml Eppendorf tubes
30. Add 700 µl ice cold isopropanol (0.7 × volume) and gently mix by inverting tubes
31. Leave at -20°C overnight in freezer
32. Centrifuge at 11,000 rpm for 10 minutes at room temperature
33. Remove and discard supernatant
34. Add 1 ml 70% ethanol
35. Centrifuge at 11,000 rpm for 10 minutes at room temperature
36. Remove and discard supernatant
37. Open tube lid and place on clean paper tissue to air dry sample. Leave for 10 – 20 minutes

38. Add 10 - 15 μl 0.1 × TE buffer

39. Incubate tube at 50°C for 1 hour for DNA elution. Gently invert to mix from time to time

40. Proceed to quality control steps

HMW DNA quality control

1. Using a spectrophotometer (e.g., Nanodrop), obtain absorbance maxima at 230 nm (A230), 260 nm (A260), 280 nm (A280) and calculate DNA concentration (ng ul^{-1}, A260 × 50), ratio A260 / A230, A260 / A280. Optimal values are A260 / A230 ≥ 2.0, and A260 / A280 ~ 1.8

2. Using a fluorometer (e.g., Qubit), obtain the DNA concentration according to manufacturer's protocol

3. Compare concentrations obtained from spectrophotometer and fluorometer. Difference should not be more than 50%

4. Prepare dilution series of DNA (e.g., 10^{-1}, 50^{-1}, 100^{-1}, 500^{-1}) in 0.1 × TE buffer and analyze DNA fragment size using Femto Pulse (Agilent Technologies) with Genomic DNA 165 kb kit following the manufacturer's protocol. Optimal values for PacBio HiFi sequencing library input DNA are $GQN_{10kb} \geq 7$, and $GQN_{30kb} \geq 5$, and average fragment size 40-50 kb

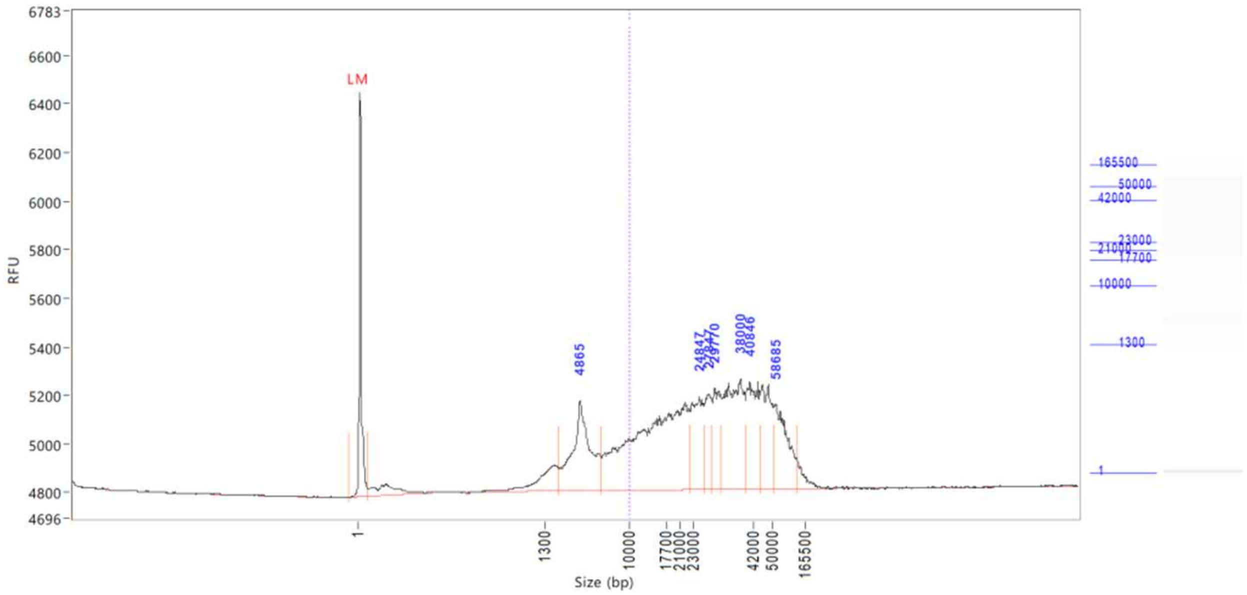

Supplementary Figure S1

Femto Pulse result for DNA obtained with lysis condition of 58°C for 30 minutes (DNA-ID: KN499). Left: line plots of distribution of DNA fragment size (bp, x-axis) and RFU (relative fluorescence unit) (y-axis). Right: corresponding gel image

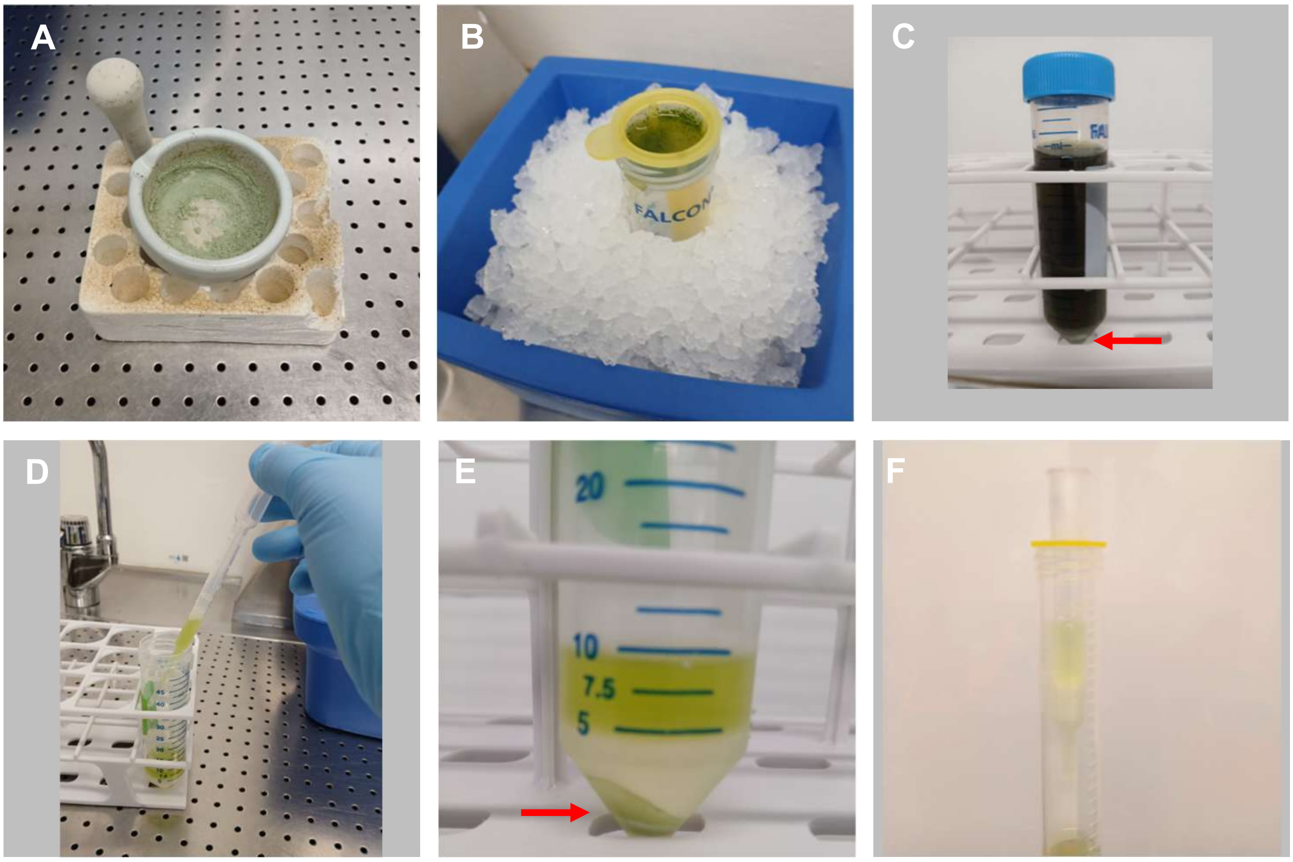

Supplementary Figure S2

Key steps for nuclei & cell isolation and Percoll™ gradient. **A.** Tissue grinding with pestle and mortar in liquid nitrogen. **B.** Filtering of tissue suspension with Corning nylon mesh pore size 100 µm. **C.** Sample centrifuging for obtaining nuclei & cell pellet (arrow). **D.** Layering sample onto a 37.5% Percoll™ gradient layer. **E.** Centrifuging Percoll™ gradient layers resulting in two fluid layers and solid nuclei & cell pellet (arrow). **F.** After CTAB lysis, pH adjustment and dilution, sample loaded on Qiagen Genomic-Tips 20/G column

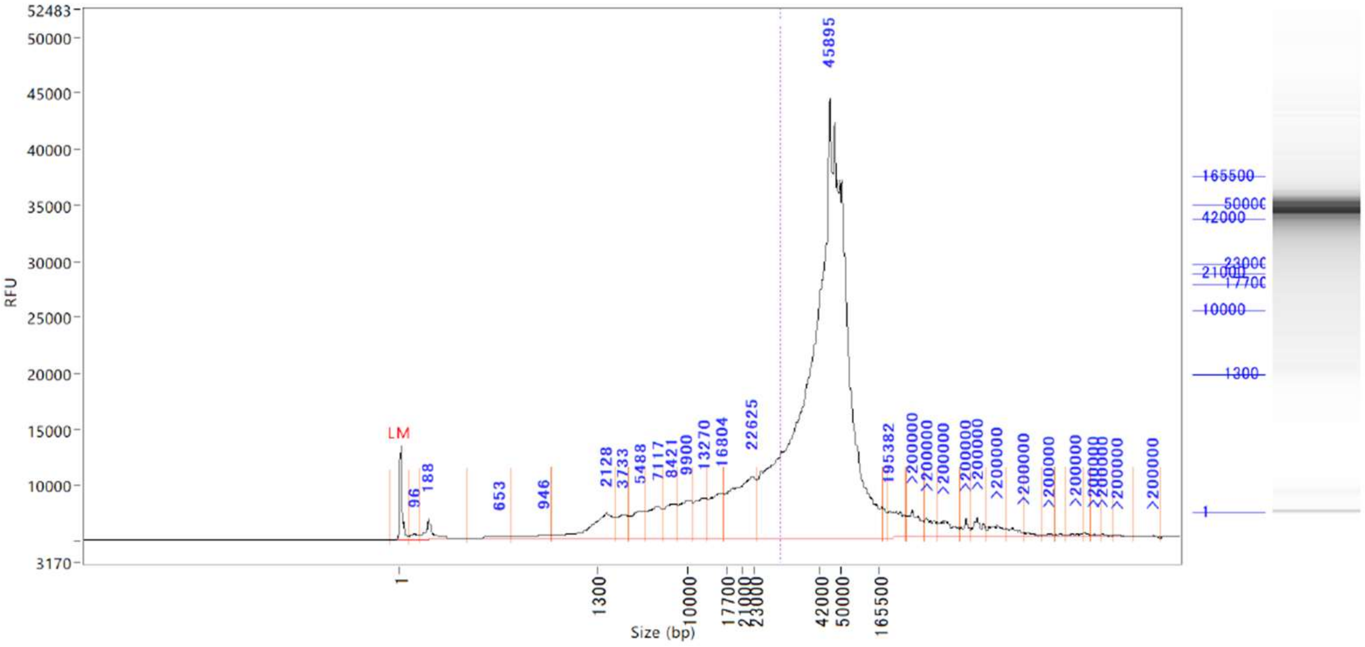

Supplementary Figure S3

Representative Femto Pulse result for *Streptocarpus schliebenii* DNA obtained using protocol established in this study (DNA-ID: KN493). Left: line plots of distribution of DNA fragment size (bp, x-axis) and RFU (relative fluorescence unit) (y-axis). Right: corresponding gel image

Supplementary Table S1 Plant materials tested in this study

Clade	Clade2	Order	Family	Taxon	RBGE accession number*Qualifier	Vaucher ID	DNA-ID
Gymnosperms		Taxales	Taxaceae	*Taxus baccata 'Fastigiata'*	19699300*B	MMOG-699	KN555
Monocots		Asparagales	Alliaceae	*Tulbaghia violacea*	19992214*A	MMOG-696	KN553
Monocots		Asparagales	Iridaceae	*Iris pseudacorus*	19694882*J	MMOG-697	KN556
Eudicots		Ranunculales	Berbaridaceae	*Epimedium perralderianum*	19694251*A	MMOG-701	KN558
Core eudicots	Rosids	Rosales	Rosaceae	*Prunus maackii*	19591737*A	MMOG-698	KN554
Core eudicots	Rosids	Geraniales	Geraniaceae	*Geranium macrorrhizum*	19890598*E	MMOG-703	KN560
Core eudicots	Asterids	Ericales	Primulaceae	*Cyclamen purpurascens*	19782439*A	MMOG-700	KN557
Core eudicots	Asterids	Boraginales	Boroginaceae	*Pulmonaria affinis*	19802831*B	MMOG-705	KN562
Core eudicots	Asterids	Lamiales	Gesneriaceae	*Corytoplectus speciosus*	20200981*A	MMOG-695	KN551
Core eudicots	Asterids	Lamiales	Gesneriaceae	*Rhynchoglossum gardneri*	19682727*A	MMOG-693	KN552
Core eudicots	Asterids	Lamiales	Gesneriaceae	*Streptocarpus schliebenii*	20131226*D	MMOG-695	See Table S2, S4, S5
Core eudicots	Asterids	Lamiales	Gesneriaceae	*Streptocarpus papangae*	19972886*AH	MMOG-688	See Table S2, S4, S5
Core eudicots	Asterids	Lamiales	Gesneriaceae	*Streptocarpus grandis*	20130764*A	MMOG-692	See Table S2, S4, S5
Core eudicots	Asterids	Aquifoliales	Aquifoliaceae	*Ilex aquifolium*	19687647*F	MMOG-704	KN561
Core eudicots	Asterids	Asterales	Asteraceae	*Erigeron pulchellus*	19780171*A	MMOG-702	KN559

Supplementary Table S2 Quality control values of *Streptocarpus* DNA extracted using different methods and conditions. Method- Nishii et al. 2023 is a previous protocol without Percoll™ gradient step; Method- This study is a new protocol developed with Percoll™ gradient in this study. PC: Percoll™, NC: Nanodrop concentration, QC: Qubit concentration

Species	Lysis time	Lysis temp.	PC (+/-)	DNA-ID	A260 / A280	A260 / A230	NC (ng μl^{-1})	QC (ng μl^{-1})	GQN_{10kb}	GQN_{30kb}	Peak bp	Sample (g)	Total vol. (μl)	Total DNA (ng)	DNA per sample (ng g^{-1})
Method- Nishii et al. 2023															
S. schliebenii	4 h	58°C	-	KN467	1.81	1.27*	15.4	11.9	9.5	8.0	64,325	16.1	240.0	2,856.0	177.8
S. schliebenii	4 h	58°C	-	KN468	1.42*	1.46*	24.4	19.7	9.6	7.8	58,124	15.5	240.0	4,728.0	305.0
Method- This study															
S. schliebenii	30 min	55°C	+	KN493	1.92	2.32	31.5	45.4	8.5	6.7	45,895	2.2	22.5	1,021.5	464.3
S. schliebenii	30 min	55°C	+	KN494	1.81	2.23	67.6	58.4	8.6	6.1	41,150	2.2	22.5	1,314.0	597.3
S. schliebenii	30 min	55°C	+	KN495	1.92	2.26	48.4	24.4	8.4	6.1	61,076	2.2	22.5	549.0	249.5
				**Average of KN493-495**	**1.88**	**2.27**	**49.2**	**42.7**	**8.5**	**6.3**	**49,373**	**2.2**	**22.5**	**961.5**	**437.0**
S. papangae	30 min	55°C	+	KN496	1.79	2.03	20.1	23.4	8.7	6.8	57,121	2.3	22.5	526.5	228.9
S. papangae	30 min	55°C	+	KN497	1.79	2.14	128.7	94.4	7.9	5.7	47,158	2.4	22.5	2,124.0	885.0
S. papangae	30 min	55°C	+	KN498	1.80	2.15	169.8	67.7	8.4	5.5	35,513	2.3	22.5	1,523.3	618.8
				**Average of KN496-498**	**1.78**	**2.11**	**106.2**	**61.8**	**8.3**	**6.0**	**46,597**	**2.3**	**22.5**	**1391.3**	**577.6**
S. schliebenii†	30 min	58°C	+	KN499	1.92	2.29	30.2	33.1	7.5	3.7*	38,000	30.8	300	9,930.0	322.4
S. schliebenii‡	30 min	55°C	+	KN507	1.86	2.35	37.6	32.1	8.8	6.5	45,201	18.2	180	5,778.0	317.5

*Indicating values with low quality or low molecular weight DNA; † Results obtained during protocol development using 58ºC lysis temperature; ‡ Results of large-scale extraction using 18.2 g of fresh leaf sample

Nishii K, Möller M, Foster RG, Forrest LL, Kelso N, Barber S, Howard C, Hart ML (2023) A high quality, high molecular weight DNA extraction method for PacBio HiFi genome sequencing of recalcitrant plants. Plant Methods 19: 41

Supplementary Table S3 Comparison between protocols for nuclei isolation and/or DNA extraction using Percoll^{TM} gradients

**Reference**	**Peterson et al. 2006**	**Folta and Kaufman 2007**	**Sikorskaite et al. 2013**	**This study**
**Plant material**	*Triticum aestivum*	*Arabidopsis thaliana*	*Nicotiana tabacum*<br>*Solanum tuberosum*<br>*Malus domestica*	*Streptocarpus* and others (see Table S1)
**Pre-treatment**	Soaked in diethyl ether	na	na	na
**Tissue grinding**	Blender	With mortar and pestle; in liquid nitrogen	With mortar and pestle; in liquid nitrogen	With mortar and pestle; in liquid nitrogen
**Extraction buffer**	1.0 M Hexylene glycol<br>10 mM PIPES-KOH (pH 6.0)<br>10 mM $MgCl_2$<br>2% PVP<br>10 mM sodium diethyldithioicarbamate	2.0 M Hexylene glycol<br>20 mM PIPES-KOH (pH 7.0)<br>10 mM $MgCl_2$<br>5 mM β-mercaptoethanol	10 mM MES-KOH (pH5.4)<br>10 mM NaCl<br>10 mM KCl<br>2.5 mM EDTA<br>250 mM sucrose<br>0.1 mM spermine<br>0.5 mM spermidine<br>1 mM DTT<br>1% PVP<br>0.1% protease inhibitor cocktail	10 mM Tris-HCl (pH 8.0)<br>10 mM EDTA (pH 8.0)<br>500 mM sucrose<br>100 mM KCl<br>4 mM Spermidine<br>1 mM Spermine<br>0.1% β-mercaptoethanol
**Filtering**	6 layers of cheesecloth	3-5 layers of cheesecloth	2 layers of cheesecloth and 1 layer of Miracloth	100 µm pore size nylon mesh

Supplementary Table S3 (continued)

Reference	Peterson et al. 2006	Folta and Kaufman 2007	Sikorskaite et al. 2013	This study
**Cell lysis**	By adding 0.5% Triton X-100 in filtrate before centrifugation	By adding 1% Triton X-100 after filtering and before centrifugation	By adding 0.5% or 1% Triton X-100 after filtering and before centrifugation	By adding 0.5% Triton X-100 after filtering and before centrifugation
**Crude nuclei collection**	800 ×*g* 20 min	na	1000 ×*g* or 1800 ×*g* 10 min	2000 ×*g* 10 min
**Gradient buffer**	0.5 M Hexylene glycol<br>10 mM PIPES-KOH (pH7.0)<br>10 mM MgCl2<br>0.5% Triton X-100<br>10 mM sodium metabisulfite<br>5 mM β-mercaptoethanol	0.5 M Hexylene glycol<br>5 mM PIPES-KOH (pH7.0)<br>10 mM MgCl2<br>5 mM β-mercaptoethanol<br>1% Triton X-100	10 mM MES-KOH (pH5.4)<br>10 mM NaCl<br>10 mM KCl<br>2.5 mM EDTA<br>250 mM sucrose<br>0.1 mM spermine<br>0.5 mM spermidine<br>1 mM DTT<br>1% PVP<br>0.1% protease inhibitor cocktail	1.0 M Hexylene glycol<br>20 mM PIPES-KOH (pH7.0)<br>10 mM EDTA<br>10 mM β-mercaptoethanol
**Percoll solution**	37.5% Percoll	30% Percoll layered on 80% Percoll layer	60% Percoll layered on 2.5 M sucrose layer	37.5% Percoll
**Gravity force**	300 ×*g* 10 min<br>650 ×*g* 10 min	2000 ×*g* 30 min	1000 ×*g* or 1200 ×*g* 30 min	1200 ×*g* 10 min
**Nuclei layer**	Bottom layer	Intermediate layer between 80% and 30% Percoll	In 60% Percoll layer	Bottom layer
**DNA extraction method**	Agarose-plug method	na	na	Modified CTAB comb. with Qiagen Genomic-tip

Folta KM, Kaufman LS (2006) Isolation of Arabidopsis nuclei and measurement of gene transcription rates using nuclear run-on assays. Nat Protoc 1: 3094–3100
Peterson DG, Tomkins JP, Frisch DA, Wing RA, Paterson AH (2006) Construction of plant bacterial artificial chromosome (BAC) libraries: an illustrated guide. Construction of plant bacterial artificial chromosome (BAC) libraries: an illustrated guide. URL: https://wheat.pw.usda.gov/jag/papers00/paper300/intro3_00.html#abstract (accessed April 12, 2025)
Sikorskaite S, Rajamäki M-L, Baniulis D, Stanys V, Valkonen JP (2013) Protocol: Optimised methodology for isolation of nuclei from leaves of species in the Solanaceae and Rosaceae families. Plant Methods 9: 31

Supplementary Table S4 Examples of DNA quality control results obtained during protocol development for *Streptocarpus schliebenii* using different Percoll™ gradient conditions. NC: Nanodrop concentration, QC: Qubit concentration

**Species**	**Percoll conc.**	**Reference**	**Lysis time & temp.**	**DNA-ID**	**A260 / A280**	**A260 / A230**	**NC (ng µl^{-1})**	**QC (ng µl^{-1})**	**GQN_{10kb}**	**GQN_{30kb}**	**Peak bp**	**Sample (g)**	**Total vol. (µl)**	**Total DNA (ng)**	**DNA per sample (ng g^{-1})**
S. schliebenii	60% Percoll; 2.5 M sucrose	Sikorskaite et al. 2013	2 hours 58 °C	KN467	1.81	4.31	7.29	1.88	nt[a]	nt	nt	4	30	56	14
S. schliebenii	80% Percoll; 30% Percoll	Folta and Kaufmam 2007	2 hours 58 °C	KN472	1.61	1.82	18.32	11.4	7.2	4.0	37,547	3	12	137	46
S. schliebenii	37% Percoll[b]	Peterson et al. 2006	2 hours 58 °C	KN482	1.80	2.44	73.52	85.1	5.8	1.0	19,974	10	72	6127	613

a: nt: not tested, b: diethyl ether treatment, blender grinding, buffer composition, and centrifugation condition were from Peterson et al. 2006

Folta KM, Kaufman LS (2006) Isolation of Arabidopsis nuclei and measurement of gene transcription rates using nuclear run-on assays. Nat Protoc 1: 3094–3100
Peterson DG, Tomkins JP, Frisch DA, Wing RA, Paterson AH (2006) Construction of plant bacterial artificial chromosome (BAC) libraries: an illustrated guide. Construction of plant bacterial artificial chromosome (BAC) libraries: an illustrated guide. URL: https://wheat.pw.usda.gov/jag/papers00/paper300/intro3_00.html#abstract (accessed April 12, 2025)
Sikorskaite S, Rajamäki M-L, Baniulis D, Stanys V, Valkonen JP (2013) Protocol: Optimised methodology for isolation of nuclei from leaves of species in the Solanaceae and Rosaceae families. Plant Methods 9: 31

Supplementary Table S5 Quality control values of DNA obtained from *Streptocarpus grandis* with or without Percoll™ and 30 min lysis time. PC: Percoll™, NC: Nanodrop concentration, QC: Qubit concentration

**Species**	**Lysis time**	**Lysis temp.**	**PC (+ / -)**	**DNA-ID**	**A260 / A280**	**A260 / A230**	**NC (ng µl^{-1})**	**QC (ng µl^{-1})**	**GQN_{10kb}**	**GQN_{30kb}**	**Peak bp**	**Sample (g)**	**Total vol. (µl)**	**Total DNA (ng)**	**DNA per sample (ng g^{-1})**
S. grandis	30 min	55°C	+	KN539	1.81	2.04	30.4	25.3	8.6	6.7	84,108	2.0	20	506	253
S. grandis	30 min	55°C	+	KN540	1.79	1.83	20.0	20.1	8.4	6.0	107,750	2.0	20	402	201
S. grandis	30 min	55°C	+	KN541	1.8	1.78	22.3	19.9	8.3	6.9	136,194	2.0	20	398	199
			**Average of KN539-541**		**1.8**	**1.88**	**24.2**	**21.8**	**8.4**	**6.5**	**109,351**	**2.0**	**20**	**435**	**218**
S. grandis	30 min	55°C	-	KN542	1.73	1.94	13.2	12.6	7.4	5.0	127,517	2.0	20	252	126
S. grandis	30 min	55°C	-	KN543	1.94	2.1	21.7	23.1	8.3	6.8	105,165	2.0	20	462	231
S. grandis	30 min	55°C	-	KN544	1.74	2.1	35.1	32.8	8.3	6.5	106,001	2.0	20	656	328
			**Average of KN542-544**		**1.80**	**2.0**	**23.3**	**22.8**	**8.0**	**6.1**	**112,894**	**2.0**	**20**	**456**	**228**

Supplementary Table S6 Quality control values of DNA obtained from a wide range of plants. PC: Percoll^{TM}, NC: Nanodrop concentration, QC: Qubit concentration

**Species**	**DNA-ID**	**A260 / A280**	**A260 / A230**	**NC (ng μl^{-1})**	**QC (ng μl^{-1})**	**GQN_{10kb}**	**GQN_{30kb}**	**Peak bp**	**Sample (g)**	**Total vol. (μl)**	**Total DNA (ng)**	**DNA per sample (ng g^{-1})**
Taxus baccata 'Fastigiata'	KN555	1.79	1.96	11.5	5.8	0	0	2,270	2.5	20	116	46.4
Tulbaghia violacea	KN553	1.83	1.70	99.1	74.5	9.0	7.3	68,605	2.1	20	1490	709.5
Iris pseudacorus	KN556	1.73	1.96	123.1	86.0	8.4	7.1	80,232	2.3	20	1720	747.8
Epimedium perralderianum	KN558	1.74	4.17	11.4	7.3	3.8	2.0	17,700	2.3	20	146	63.6
Prunus maackii	KN554	2.12	0.68	8.5	0.7	0	0	3,240	2.1	20	14	6.7
Geranium macrorrhizum	KN560	1.47	4.02	12.0	7.7	6.7	4.7	137,056	2.3	20	154	67.0
Cyclamem purpurascens	KN557	1.77	1.50	111.0	109.8	9.3	8.5	99,131	2.2	20	2,196	998.2
Pulmonaria affinis	KN562	1.66	2.43	7.27	4.8	8.5	6.9	114,646	2.2	20	96	43.6
Corytoplectus speciosus	KN551	1.91	2.17	70.8	55.3	7.6	5.1	60,078	2.2	20	1,106	502.7
Rhynchoglossum gardneri	KN552	1.89	1.02	39.1	17.2	6.5	4.7	79,457	2.3	20	344	149.6
Ilex aquifolium	KN561	1.80	2.00	117.0	199.2	8.2	6.5	42,824	2.4	20	3,984	1,660
Erigeron pulchellus	KN559	1.75	1.51	174.0	143.9	8.9	7.8	65,516	2.1	20	2,878	1370.5
